# Effects of peroxidase and superoxide dismutase on physicochemical stability of fish oil-in-water emulsion

**DOI:** 10.1038/s41538-022-00146-2

**Published:** 2022-06-23

**Authors:** Lijing Ke, Ying Tan, Yang Xu, Guanzhen Gao, Huiqin Wang, Sihao Luo, Jianwu Zhou, Qiang Wang

**Affiliations:** 1 Food Nutrition Science Centre, School of Food Science and Biotechnology, Zhejiang Gongshang University, Hangzhou, China; 2 Institute of Food Science and Technology, Chinese Academy of Agricultural Sciences, Beijing, China

**Keywords:** Biochemistry, Food nanotechnology

## Abstract

How to maintain the physicochemical stability of oil emulsion has been one of the major challenges in food industry. Previously we reported the demulsification effects of catalase in the fish oil emulsion. In comparison, the influences of other two metal ion-containing oxidoreductases, horseradish peroxidase (HRP) and copper/zinc superoxide dismutase (SOD), on the emulsion’s stability were investigated. Submicron fish oil-in-water emulsion stabilized by polysorbate 80 was prepared by high-speed homogenization. Its physical stability was evaluated by visual and microscopic observation, turbidity and light scattering measurements, while chemical stability by the hydroperoxide content and lipid peroxidation. HRP demulsified the emulsion in a concentration-responsive manner after 3–7 days’ incubation, resulting in a decreased turbidity and significant delamination. The enlargement of oil-polysorbate droplets and protein precipitates were confirmed by size distribution and TEM observation. HRP initially elevated the emulsion’s hydroperoxide then decreased it while raising TBARS levels during 7-Day incubation. In contrary, SOD stabilized the emulsion physically and chemically. The demulsification was correspondingly attributed to the oxidation catalyzing activity of the peroxidase and the electrostatic and hydrophobic interaction between lipids and proteins. This study adds new insight to the influences of the two oxidoreductases on the stability, lipids and peroxides of food emulsions, proposes an exciting subject of elucidating the underlying mechanism.

## Introduction

Fish oil is rich in ω−3 polyunsaturated fatty acids, which are so susceptible to environmental conditions and easily discomposed or oxidized^1^. It is reported that emulsion can improve bioavailability of lipid products^2^. Thus, how to maintain the stability of emulsion has drawn increasing attention.

The effect of enzymes on the stability of emulsion models of lipid foods has been widely studied. Hydrolases such as lipase, chymotrypsin, and trypsin are the most extensively studied enzymes in emulsions^3^. For example, emulsion stabilized by trypsin hydrolysates can delay the lipids oxidation and lengthen shelf life^4^. Phospholipase and protease can break the emulsion formed by aqueous extraction processing to extract edible oil^5^.

The influences of oxidoreductase on food emulsions have been reported, too. The copper-zinc superoxide dismutase (SOD) exhibited antioxidant effects in Fe^2+^ accelerated oxidation of milk fat system and improved the stability of milks rich in linoleic acid. The superoxide dismutase is more effective in conjunction with catalase, but could not inhibit the hemin-catalyzed oxidation of emulsions containing 23% (w/w) soybean oil^6^. In contrast to SOD, the sole use of heme containing catalase caused a fast demulsification of the fish oil-polysorbate 80 emulsion^7^. To elucidate and compare the effects of metal ion-containing oxidoreductases on the fish oil emulsion, horseradish peroxidase (HRP) and copper/zinc-SOD were investigated in this study. HRP was chosen because it contains heme, alike CAT. The SOD was chosen as it may possess antioxidant and stabilizing effects on the fish oil emulsion.

Horseradish peroxidase (HRP) is a kind of peroxidases, which catalyzes the hydrogen peroxide induced oxidation of organic substrates, and one of the most popular heme proteins in plants. It has already been used as a model enzyme due to its commercial production in large scale^8^. It is stable over a wide pH and temperature range and versatile to be used in various media other than the aqueous solution^9^. As the most heat-stable enzymes in the plants, the active HRP could be expected to exist in many raw and processed foods. It has been used as an index of blanching fruits and vegetables, as well as sterilization^10^.

Superoxide dismutase (SOD) is another important oxidoreductase commonly found in all kinds of living aerobic organisms. SOD catalyzes the dismutation of superoxide radicals (O_2_^·−^) to molecular oxygen (O_2_) and hydrogen peroxide^11^, which could be used by HRP for oxidizing the substrates. It is often employed as an antioxidative agent in food to retard the overall oxidation^12^. Different from HRP, SOD has three isoforms containing different metal ions, i.e. copper, zinc and manganese.

Being the common composition of raw and processed foods, these two metalloenzymes often encounter oil emulsions containing significant amount of unsaturated fats, may subsequently affect the physical and chemical characteristics of emulsions via non-covalent or covalent interactions. Such influences are still largely unknown. Therefore, it is worth of elucidating the influences of HRP and Cu,Zn-SOD on the physicochemical stability of emulsions, especially when the demulsifying effects of catalase have been reported recently^7^.

In this study, fish oil-in-water submicron emulsion, using polysorbate 80 as emulsifier, was prepared by high-speed homogenization. The interactions between horseradish peroxidase, superoxide dismutase and oil-emulsifier droplets were studied, using the emulsion as a model system, to illustrate the implicit factors that may project influence on the quality and stability of food products rich in unsaturated lipids.

## Results and discussion

The average hydrodynamic diameter (D_h_) of fish oil-polysorbate 80 emulsion droplets (FOE, 5‰) was 130.6 nm and ζ-potential of which was around −20 mV. The major granular parameters were consistent with the previous work^1,7^.

### Visual examination, turbidity and enzymatic activities of emulsions

**Fig. 1:**
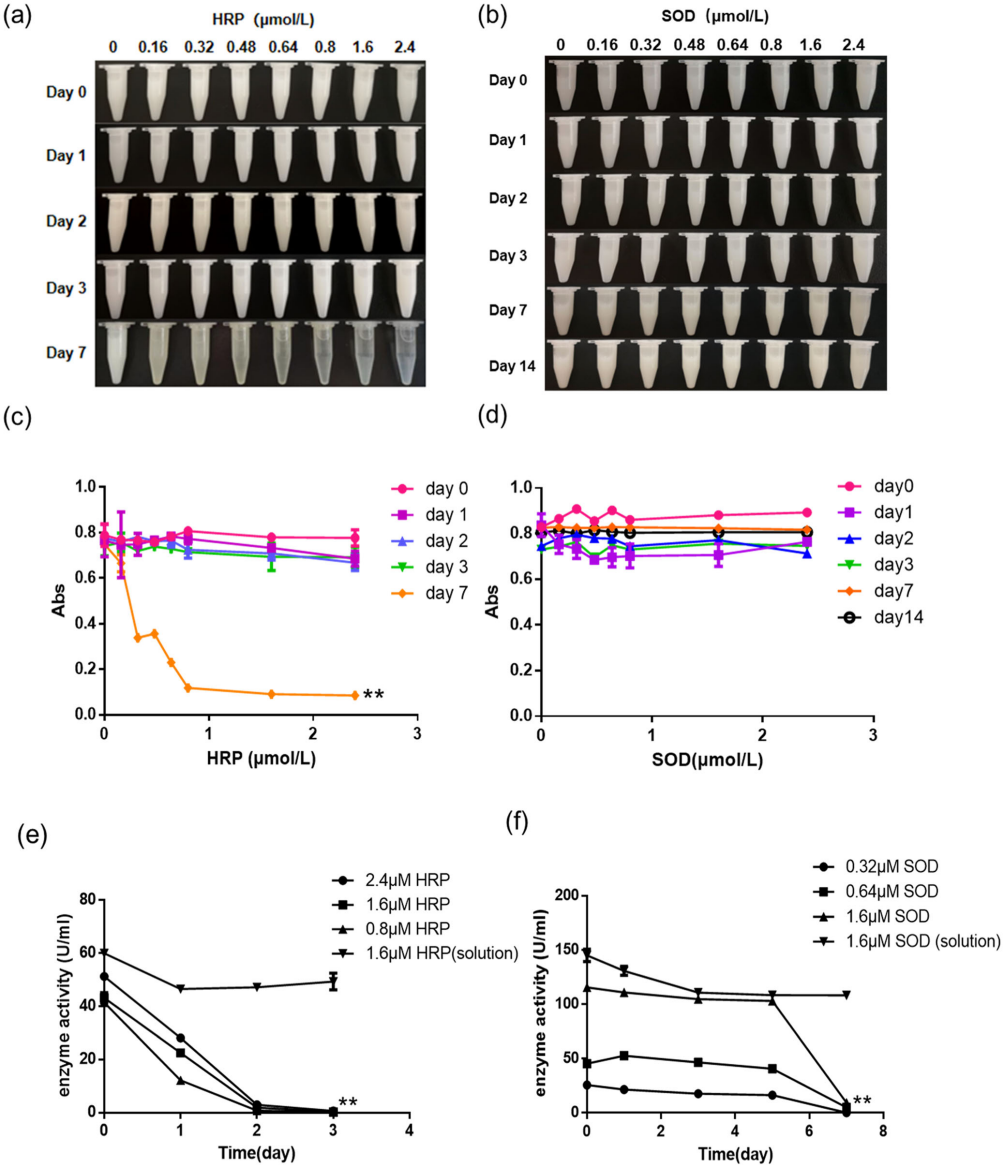
Visual observation and turbidity of FOE in the presence of HRP and SOD. **a** FOE + HRP; (**b**) FOE + SOD; (**c**) turbidity of FOE + HRP; **: differences were highly significant before and after incubation, *p* < 0.01; (**d**) turbidity of FOE + SOD; (**e**) enzyme activity of HRP in FOE and solution, **: differences were highly significant between HRP aqueous solution and HRP dissolved in the FOE, *p* < 0.01; (**f**) enzyme activity of SOD in FOE and solution, **: differences were highly significant between SOD aqueous solution and SOD dissolved in the FOE, *p* < 0.01. The error bars represent standard deviation.

As shown in the Fig. 1a, the emulsion aliquots in the presence of HRP did not show any visible changes in FOE during the first three days of incubation at 37 °C. Phase separation and precipitation were observed four days later (on Day 7). The emulsion’s cloudiness gradually faded as its transparency increased along the increasing HRP concentrations. It is in good agreement with the turbidity measurement, as shown in Fig. 1c. The fact that precipitates increased as demulsification progressed in the emulsion, indicates an interaction between HRP and the FOE droplets. The turbidity dropping was attenuated when the enzyme’s concentration was higher than 0.8 µM. The growing number of HRP molecules accumulated on the oil droplets’ surface would increase the visible light scattering and result in a higher turbidity.

Superoxide dismutase (SOD) was added to the emulsions at the same molar concentration as HRP. As shown in the Fig. 1b, there was neither demulsification nor precipitation in the presence of SOD during the 14-day incubation at 37°C, which was consistently with their turbidity measurements (Fig. 1d). It confirmed the explicit differences between the effects of HRP and SOD on the emulsion.

The almost spontaneous occurrence of enzyme deactivation, demulsification and protein precipitation was found in the interaction between catalase and the fish oil emulsion^7^. To verify whether the similar pattern of interaction could be applicable to the HRP/SOD in the same type of emulsion, the enzymatic activities of emulsions and their pure enzyme aliquots (at 1.6 µM) were determined as function of incubation time. As shown in Fig. 1e, the HRP activity of the pure enzyme aliquots decreased slightly after the first day and then remained the same. In contrast, the HRP activity of FOE added with the enzyme dropped rapidly in the first two days and eventually diminished on Day 3. The HRP activity loss in FOE occurred earlier than the visible demulsification or protein precipitation, which is different from the profile of catalase-emulsion system, suggesting the inactive HRP may still interact with oil droplets and induce the flocculation later.

The enzymatic activity of SOD solution gradually and slightly declined during the first three days and then stay unchanged during the rest four days. The SOD activities of FOE decreased slightly during the first five days, followed by a sudden drop losing more than 90% of activity on Day 7 (Fig. 1f), which could be attributed to the inactivation caused by lipid hydroperoxides in emulsions^6^. However, unlike HRP, the inactivation of SOD did not result in the compromised turbidity of FOE.

**Fig. 2:**
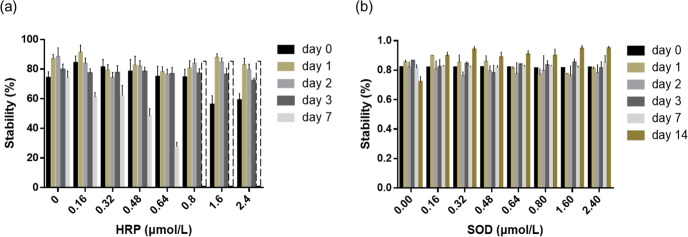
Effects of HRP and SOD on the stability of fish oil emulsion. **a** FOE + HRP; (**b**) FOE + SOD. The dash line column indicates that the stability measurement of FOE + HRP on day 7 at HRP concentrations ranged from 0.8 to 2.4 µM was not reliable due to the already occurred demulsification and precipitation of HRP. The error bars represent standard deviation.

The physical stability of FOE was determined by comparing the turbidity before and after centrifuge, to avoid the possible artifacts caused by uneven sampling. The stability of FOE with different concentrations of HRP fluctuated slightly but insignificantly on Day 0, except the decrease at the relatively high HRP concentration of 1.6 µM and 2.4 µM. The stability remained unchanged within the first three days at all HRP concentrations but started to drop on Day 7. The emulsion stability gradually declined in a dose-dependent manner till the concentration reached 0.64 μM. The turbidity could not be measured accurately at the higher HRP concentrations due to the poor light absorbance caused by demulsification and precipitation (Fig. 2a). In contrast, the addition of SOD did not change the emulsion stability at all assayed concentrations (Fig. 2b).

Proteins have been demonstrated to stabilize oil-in-water emulsions. For instance, the whey protein isolates stabilized microemulsion and nano-emulsion of corn oil^13^, and β-lactoglobulin kept *n*-hexadecane emulsions stable^14^. Such stabilizing effects were attributed to the distribution of proteins on the water/oil interface of oil droplets. However, SOD may act differently compared to those protein emulsifiers, as SOD might not attach to the oil/water interface. Otherwise, the ζ-potential of emulsion would have changed right after the addition of SOD, as described below.

### Impacts of HRP and SOD on emulsion particle size andζ-potential

**Fig. 3:**
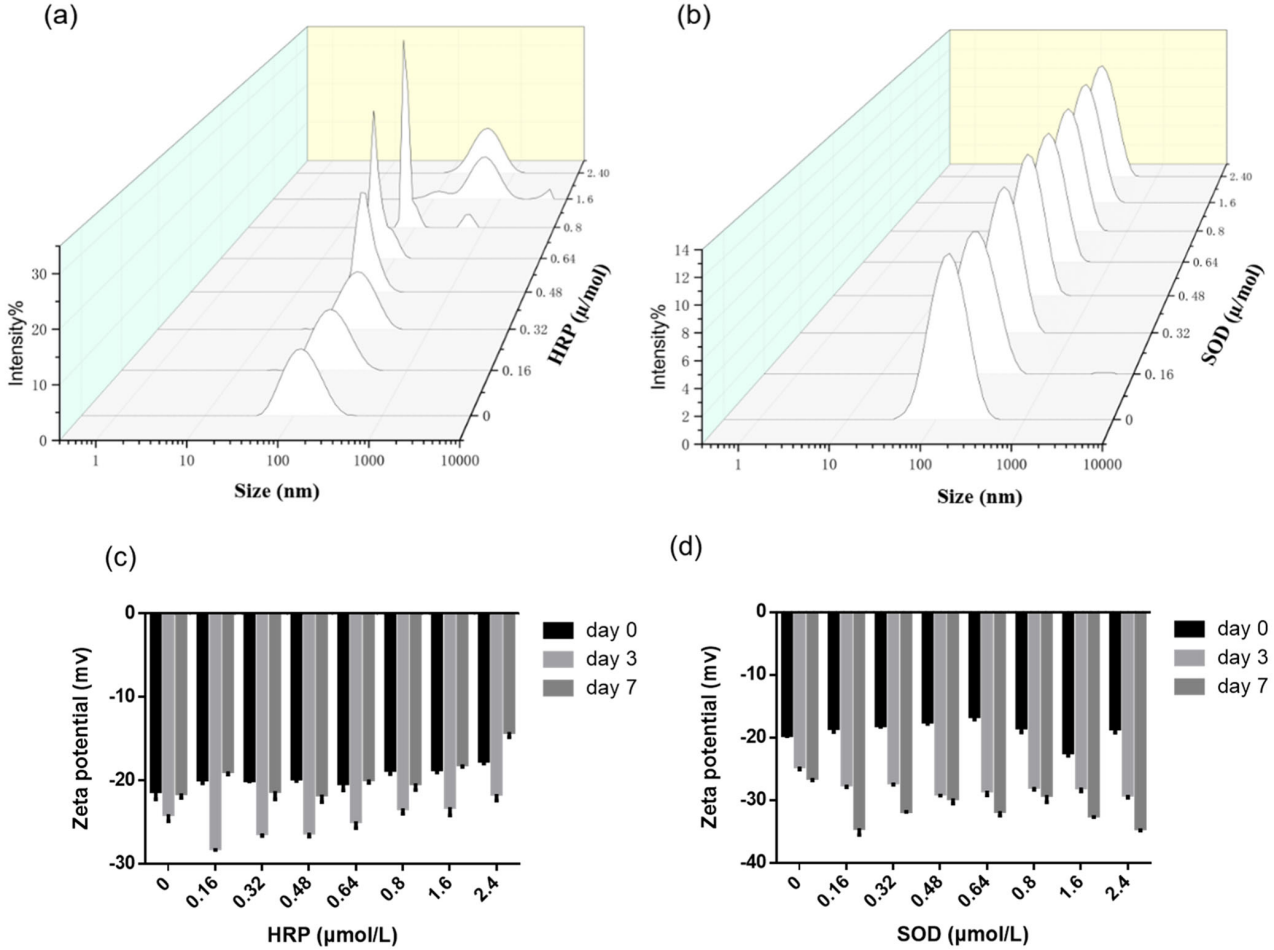
Granular properties of oil-in-water submicron emulsion of fish oil. **a** size distribution of FOE in the presence of HRP on Day 7; (**b**) size distribution of FOE in the presence of SOD on Day 7; (**c**) ζ-potential of FOE in the presence of HRP on Day 0, Day 3, and Day 7; (**d**) ζ-potential of FOE in the presence of SOD on Day 0, Day 3, and Day 7. The error bars represent standard deviation.

The impacts of HRP and SOD on the granular properties and surface charges of droplets in the emulsion were examined. As shown in Fig. 3a, the FOE droplets exhibited a single size distribution from 100 nm to 300 nm. After seven days of incubation, the particle size distribution of FOE did not change by HRP at its low concentrations, e.g., 0.16 µM and 0.32 µM (Supplementary Table 1). The scattering light gradually intensified as the HRP concentration increased, till the major droplet size distribution shifted to micrometers at the HRP concentration higher than 1.6 µM. The emulsion demulsified in due course.

Meanwhile, there was no obvious changes in FOE’s ζ-potential at the low and medium HRP concentrations on Day 0 (Fig. 3c), except mild decrease at the high HRP concentrations, and the significant increase on Day 3 in presence of HRP. As its isoelectric point is close to 9, the HRP carries positive charge in the slightly acidic FOE emulsion (pH ≈6.0). The mildly decreased ζ-potential is in good agreement with HRP’s positive charge. In contrast, the ζ-potential of such emulsion decreased on Day 7, particularly at 2.4 µM of HRP, echoing the destructive effects of HRP on FOE. Notably, this ζ-potential is unlikely reporting the static charge of fish oil lipid droplets, which has been demulsified by now, but more likely that of the polysorbate 80 colloidal particles. Furthermore, the increased ζ-potential on Day 3 is most likely attributed to the lipid peroxidation catalyzed by HRP and the subsequent higher level of carboxyl. The higher ζ-potential of control emulsion on Day 3 and Day 7 is probably caused by the same reason.

SOD neither significantly alter the droplet size of FOE (Fig. 3b, Supplementary Table 2), nor the ζ-potential on Day 0. However, it did increase the ζ-potential at all concentrations on Day 3 and Day 7 (Fig. 3d). The elevated ζ-potential is consistent with the emulsion stabilizing effects of SOD, but lack of the clear causes. The SOD molecules neither bind to the emulsion droplets, otherwise the ζ-potential on Day 0 would have changed, nor promoted the lipid peroxidation. The low peroxidation products level rules out the possibility of increased carboxyl level. It would acquire follow-up study to illuminate whether the enzyme’s dismutation activity contributes to the higher static charge of lipid droplets.

### TEM observation of emulsion micromorphology

**Fig. 4:**
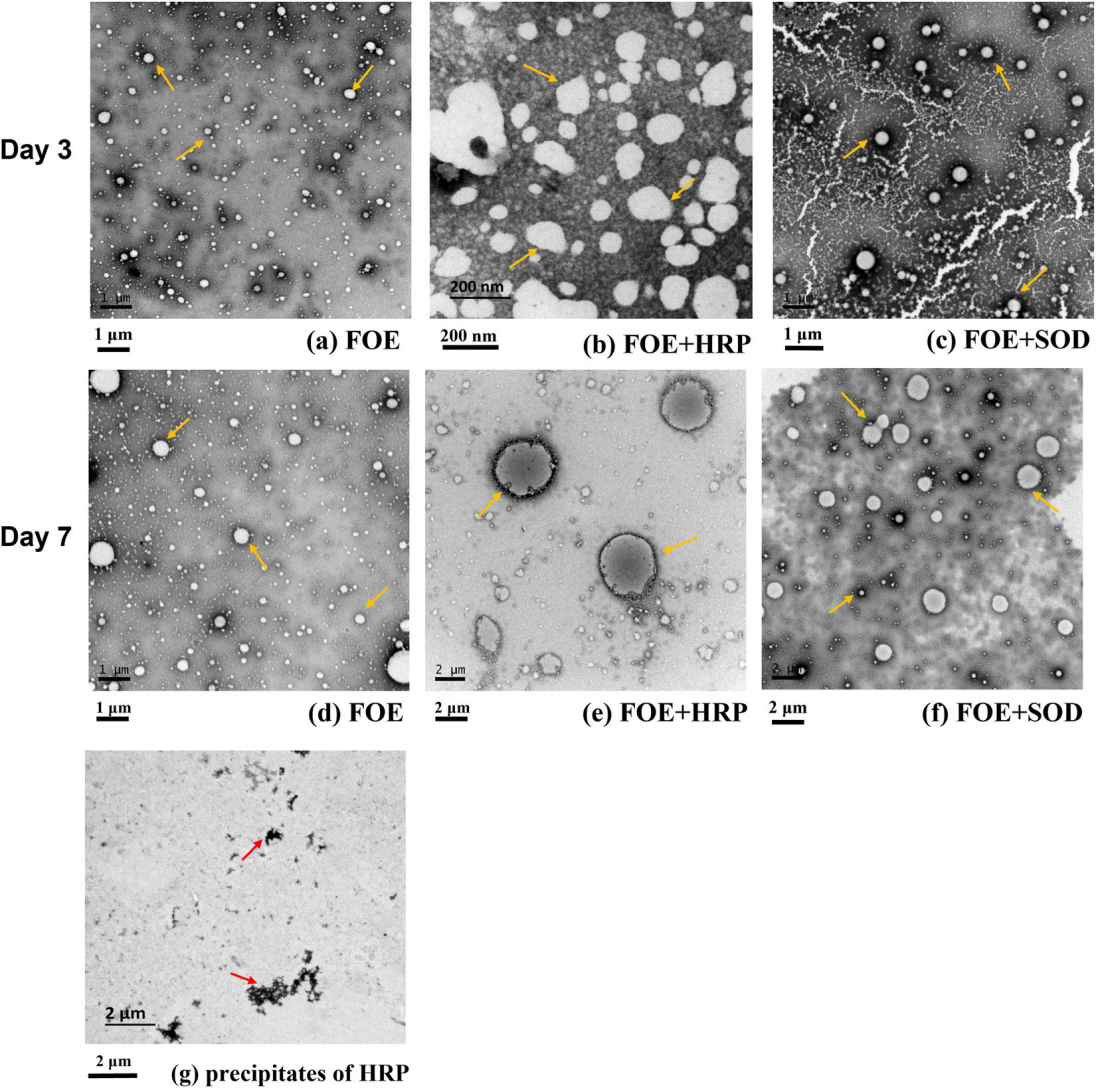
TEM observation of fish oil emulsion in the presence of HRP and SOD. Yellow arrows indicate fish oil droplets. Red arrows indicate precipitates of HRP. Diagram (**a**) FOE droplets on Day 3 (Scalebar 1 µm); (**b**) droplets in FOE + HRP sample on Day 3 (Scalebar 200 nm); (**c**) droplets in FOE + SOD sample on Day 3 (Scalebar 1 µm); (**d**) FOE droplets on Day 7 (Scalebar 1 µm); (**e**) microdroplets emerged in FOE + HRP sample on Day 7 (Scalebar 2 µm); (**f**) droplets in FOE + SOD sample remained the same on Day 7 (Scalebar 2 µm); (**g**) the HRP precipitates in FOE + HRP sample on Day 7 (Scalebar 2 µm).

The TEM observation confirmed the demulsifying effects of HRP and subsequent enlargement of oil-polysorbate droplets, which were agreed with the turbidity and dynamic light scattering measurements. After three days of incubation, the size of droplets ranged from a few dozen to one hundred nanometers in the absence of HRP, as shown in Fig. 4a. In the presence of HRP, the nanodroplets began to aggregate and emerged into submicron droplets (Fig. 4b). After 7 days of incubation, the FOE droplet stayed almost the same (Fig. 4d), while in the HRP aliquots, micron-sized droplets were observed to be surrounded by layers of HRP (Fig. 4e, HRP appeared to be in darker color). The precipitates with irregular chain-like shape were found in the FOE + HRP emulsion (Fig. 4g). They are quite similar to the precipitates we observed previously in the catalase added FOE^7^, only less condensed and smaller. Unlike HRP, in the presence of SOD, the droplets remained in nano- and submicro-sized, possibly surrounding by the enzyme (Figs. 4c, f). Despite the mild increase in the droplet size on Day 7, no protein precipitates were observed.

### Impacts of HRP and SOD on lipid oxidation

**Fig. 5:**
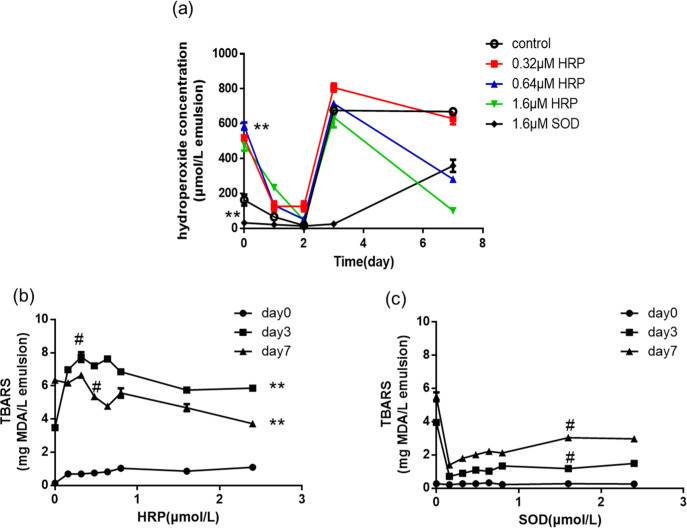
Lipid oxidation and peroxidation levels in FOE. **a** primary lipid oxidation indicated by hydroperoxide content of FOE in the presence or absence of HRP, **: differences were highly significant between control (FOE alone) and HRP/SOD added emulsions on Day 0, *p* < 0.01; (**b**) lipid peroxidation in FOE + HRP on Day 0, Day 3 and Day7, #: differences were significant between different HRP concentration, *p* < 0.05; **: differences were highly significant between incubation time length, *p* < 0.01; (**c**) lipid peroxidation in FOE + SOD on Day 0, Day 3 and Day7, #: differences were significant between different SOD concentration, *p* < 0.05. The error bars represent standard deviation.

As the lipid oxidation initiates and progresses, hydroperoxides are produced by the oxidation of polyunsaturated fatty acids, triggered by superoxide radicals in surrounding microenvironment and catalyzed by the exerted ferrous iron. The hydroperoxide content could be determined by Fenton reaction. As shown in Fig. 5a, hydroperoxide was found to be rather low (<200 µM) in the FOE on Day 0 in the absence of HRP, and declined to below 30 µM after 2 d, followed by the dramatical increase on Day 3 and stayed at the same level on Day 7. The content of hydroperoxide in the aliquot of HRP-added emulsion increased significantly on day 0 by nearly three times. Even at its low concentration (0.32 µM), HRP catalyzed the formation of hydroperoxide (>500 µM). The HRP-added aliquots exhibited similar trends as the emulsion control, except the significant drops in the hydroperoxide content in the presence of 0.64 µM and 1.6 µM HRP on Day 7. On the contrary, the presence of SOD (1.6 µM) immediately reduced hydroperoxide on Day 0, efficiently inhibited the formation of hydroperoxide up to 80% during the first three days and retained hydroperoxide level to half of the control’s on Day 7. The inhibitory effects of SOD weakened on Day 7, which is because the enzyme had lost most of its activity.

Antioxidant proteins and peptides were reported to mitigate the peroxidation of fats and oils, particularly those rich in polyunsaturated fatty acids^15,16^. The lipid peroxidation level of FOE was determined by measuring the content of thiobarbituric acid reactive substances (TBARS). Theoretically horseradish peroxidase should promote lipid peroxidation as it catalyzes oxidation of substrates triggered by hydrogen peroxide or other organic peroxide, such as hydroperoxide in the FOE. The addition of HRP promoted the peroxidation of FOE on Day 0 and Day 3 (Fig. 5b), but decreased the TBARS level on Day 7 at the enzyme concentrations higher than 0.32 µM. On Day 3, TBARS level reached the maximum at the HRP concentration of 0.32 µM, then started to decline at the concentrations of 0.64 µM and higher. This is quite different from the effects of adding CAT, an antioxidant enzyme, to the same type of FOE^7^, reflecting the opposite role the two enzymes play in FOE peroxidation. The effects of HRP on the TBARS level were synchronized with those on the hydroperoxide content.

In comparison, SOD effectively inhibited the lipid peroxidation at all the assayed concentrations, although the higher SOD concentration did not exhibit stronger inhibitory effects (Fig. 5c). In fact, the high concentrations of SOD (e.g. 1.6 and 2.4 µM) resulted in slightly higher TBARS level than the aliquots at the lower concentrations (e.g. 0.16–0.8 µM). While the exact reason behind this warrant further studies, one possible cause may be that more SOD could produce more hydrogen peroxide which in turn accelerate the lipid peroxidation^6^.

### General discussion

It is known that HRP promotes oxidation of linoleic acid by the cleavage of hydroperoxide^17^. In this study, however, the initiate level of hydroperoxide in FOE was low, which was then increased by HRP almost instantly (as shown in Fig. 5a, Day 0). This unexpected increase in the hydroperoxide could be attributed to the ferrous ions in heme of HRP, which catalyzed alkoxyl radicals’ generation. The alkoxyl radicals react rapidly with the fish lipids and produce hydroperoxides in a chain reaction^18,19^. The newly generated hydroperoxide was then used to promote oxidation of unsaturated fats by HRP and therefore gradually decreased on Day 1 and Day 2. Such trend ended on Day 3 when HRP deactivated, and hydroperoxide level raised again. On the other hand, the dramatical increase in hydroperoxide of pure FOE on Day 3, in absence or at the low concentration of HRP, should be the result of accumulated free radicals generated by lipid autoxidation. It has also been reported that H_2_O_2_ could be generated in an aqueous suspension of lipid hydroperoxide^20^. The lipid hydroperoxide declined again in FOE with medium and high concentration of HRP on Day 7, which could be well explained by the demulsification that reduced the total amount of lipids dispersed in the liquid phase.

Furthermore, as mentioned earlier, horseradish peroxidase started to demulsify FOE after three days of incubation while SOD did not cause such change but stabilized FOE. Simultaneously, HRP raised up the TBARS levels on Day 3, indicating the co-occurrence of demulsification and lipid peroxidation. Interestingly, the enzyme lost all its activity in due course. After seven days’ incubation, the demulsification progressed and became visible with bare eyes, while the TBARS values decreased, and the enzyme precipitated. However, in absence of HRP, the FOE remained physically stable although its lipid peroxidation is like that of HRP aliquots or even more severe. It is consistent with the earlier report that lipid peroxidation was not the sufficient condition for the catalase-induced demulsification of fish oil emulsions^7^. Therefore, HRP may share a similar mechanism of destabilizing oil emulsions with catalase. The longer time HRP acquired to demulsify the emulsion (seven days), compared to the catalase (1 day), could be correlated to two factors: (1) one fourth of heme number at the same protein molar concentration as catalase;^21,22^ (2) HRP is smaller and less hydrophobic than catalase.

It has been known that heme proteins, such as hemoglobin and myoglobin, can initiate peroxidation of polyunsaturated fatty acids (PUFAs) by triggering a chain reaction of free radicals^23^. Horseradish peroxidase and catalase are both peroxidases and heme proteins, containing ferric iron (Fe (III)) in the resting state. The two enzymes caused similar demulsification effects and increased lipid peroxide level, although at different speed. Electrons from hydrogen peroxide or organic substrate, i.e. PUFAs in the FOE, oxidize the peroxidases to a powerful oxidizing agent namely compound I containing oxyferryl heme (Fe^4+^=O). Compound I will be reduced back to the native heme-(Fe(III)) state via either one-electron or two-electron transfer reaction^21,24^. Therefore, the peroxidase can promote peroxidation in fish oil emulsion and electron transfer between alkyl, alkoxyl, carboxyl groups of the lipids and oxygen and free radicals in the lipid/water interfacial space.

Polysorbate 80 was added to stabilize the oil/water interface of fish oil emulsion. As we argued previously^7^, the competitive binding of polysorbate by protein is unlikely the reason of HRP-induced demulsification as well, because HRP would otherwise have remained soluble in the aqueous phase. The direct contact of HRP with lipids may offer a reasonable explanation for its faster deactivation and precipitation in the emulsion than in their aqueous solution, possibly driven by the conformational changes of proteins on the water/lipid boundary.

Apart from illuminating HRP’s vital influences on the physical and chemical stability of the emulsions rich in PUFAs, this study also elucidated the potential of superoxide dismutase as an efficient stabilizing agent in such emulsion. In addition, the species and evolution of radicals involved in the heme-catalyzed demulsification and lipid peroxidation would be of particular interest to food emulsion studies. Although the exact mechanism of this enzyme triggered demulsification remains unclear, we argue that it may involve interplays between the hydrocarbon chains of lipids and the hydrophobic domains of enzymes. The hydroperoxyl radicals or hydroperoxide-like molecules on the oil/water interface are more likely the ‘substrates’ of the enzyme. It has been hypothesized that the large surface potential of microdroplets may promote the oxidation of hydroxide ion to form hydroxyl radicals^25^. A similar mechanism may be proposed for the oxidoreductase-triggered demulsification in the submicron emulsion of fish oil and polysorbates. The catalase was the first case, and now the horseradish peroxidase. Some other factors, i.e., types of surfactants, ratio of saturated and unsaturated fatty acids, ion and pH, would significantly affect the enzymatic activities and electron transfer at the oil/water boundary and subsequently regulate the physicochemical stability of fish oil emulsion, therefore ought to be studied in future.

In conclusion, we demonstrated horseradish peroxidase but not superoxide dismutase initiated and accelerated the demulsification of polysorbate 80 stabilized oil-in-water submicron fish oil emulsion. HRP deactivated and precipitated spontaneously. The peroxidase’s heme/iron, and the electrostatic and hydrophobic interaction between lipids and proteins, are believed to play the corresponding roles in the demulsification. The redox interplay between HRP and hydroperoxide or hydroperoxyl radicals on the oil/water interface promoted lipid peroxidation, shorten the hydrocarbon chain of PUFAs, and subsequently make the lipid/polysorbate assembly more vulnerable to the disturbance of HRP. SOD, in contrary, inhibited the lipid peroxidation and maintained the physical stability of emulsion. This study highlights the influences of the two oxidoreductases commonly found in foods on the physicochemical stability of an unsaturated fats-rich emulsions, proposes an exciting subject of elucidating the underlying mechanism.

## Methods

### Materials

Fish oil (EPA 18.3%, DHA 12.3%), provided by Sinomega Co., Ltd., China; horseradish peroxidase (HRP, Sigma China); polysorbate 80 (Tween-80) and copper/zine superoxide dismutase ((Cu/Zn)-SOD) were purchased from Sinopharm Chemical Reagent Co., Ltd., China. All the solutions were prepared with deionized water (Milli-Q, Millipore, U.S.).

### Preparation of submicron emulsions

Polysorbate 80 was dissolved in deionized water (v/v, 1‰). Fish oil (v/v, 5‰) was added into the polysorbate 80 solution drop by drop and homogenized at 18,000 rpm for 5 min (high-speed homogenizer T18DS25, IKA, Germany) at 40°C in water bath. The fish oil submicron emulsions (FOE) (pH ≈ 6.3) were obtained by filtered with 0.22 μm PES membrane at 40°C. The concentration of HRP or SOD in the emulsion was 0 µM, 0.16 µM, 0.32 µM, 0.48 µM, 0.64 µM, 0.8 µM, 1.6 µM, 2.4 µM. All the emulsion samples were incubated at 37°C for seven days or 14 days. The influences of HRP and (Cu/Zn)-SOD on the physical and chemical stability of FOE were evaluated by visual examination, microscopic observation and determination of turbidity, stability, particle size, ζ-potential and lipid oxidation.

### Turbidity and stability

The emulsion turbidity is correlated to the droplets size and concentration. It was determined by measuring the absorbance at 600 nm (UV-5100, HITACHI, Japan) at room temperature (≈25°C) after the emulsion was diluted with deionized water by 20 times. The turbidity was presented as the average value of triple measurements.

To evaluate the emulsion stability by centrifugation test^26^, all samples were centrifuged at 3000 rpm for 10 min at room temperature. The turbidity of emulsion and the supernatant were measured to obtain *Aa* and *Ab*, respectively. The Equation 1 below was used to calculate the emulsion stability.

1 $${{{\mathrm{Stability}}}} = Ab/Aa \times 100\%$$

### Droplet size and ζ-potential

One milliliter emulsion sample was added into light scattering cuvette to determine the droplet size and ζ-potential at 25°C using dynamic light scattering (ZetaSizer Nano-ZS, Malvern Instruments Co. Ltd., Worcestershire, UK). Each measurement was performed three times after 60 s of equilibrium. The emulsions were diluted if the light scattering intensity was too high to obtain reliable data.

### Transmission electronic microscopy (TEM) observation

The emulsion sample was supplied to the 300-mesh copper grid covered with Formvar film, using filter paper to absorb excess liquid from the edge of copper mesh. The negative staining was carried out with 1% (w/v%) of phosphotungstic acid. Then the air-dried copper grid supplied for observing the surface morphology and size of the emulsion droplets on the transmission electron microscope (TEM, Joel JEM-1230, Japan) at an acceleration voltage of 80 kV.

### Measurements of horseradish peroxidase and superoxide dismutase activity

The activity of HRP were measured by spectrophotometer at room temperature (≈25°C). The HRP catalyzes 2,2’-azino-bis (3-ethylbenzothiazoline-6-sulfonic acid) (ABTS) in the presence of hydrogen peroxide to form an oxidized ABTS with increased absorbance at visible light wavelength. For determination of HRP activity, 3 mL of 1 mM ABTS solution and 0.1 mL of 2 μM H_2_O_2_ were mixed with 0.1 mL of the HRP solution or emulsion samples containing HRP. The sample’s absorbance was measured at 414 nm for 1 min. One unit of enzyme activity was defined as the absorbance at 414 nm changed 0.01 unit per minute^27^.

The SOD activity determination was based on the enzyme’s ability to inhibit the oxidation of hydroxylamine by the O_2_^·−^ produced from the xanthine-xanthine oxidase system (the amount of SOD corresponding to one SOD activity unit (U) when the SOD inhibition rate reaches 50% per milliliter of reaction solution). 50 µL of each sample was mixed with 1.0 mL of 0.06 M phosphate buffer (pH 7.8), 0.1 mL of 10 mM hydroxylamine hydrochloride, 0.2 mL of 7.5 mM xanthine, 0.2 mL of 0.2 mg/mL xanthine oxidase and 0.49 mL double-distilled water in the tube. After mixing, the tubes were incubated in 37°C water bath for 30 min. Afterward, samples were cooled naturally to room temperature and allowed to mix with 2.0 mL 0.33% p-aminobenzenesulfonic acid. Then the absorbances were measured at 535 nm^28^. The SOD activity was computed by the following formula:

2 $${{{\mathrm{SOD}}}}\;{{{\mathrm{activity}}}} = \frac{{\left( {A_{blank} - A_{sample}} \right)}}{{A_{blank}}} \div 50{{{\mathrm{\% }}}} \times {{{\mathrm{B}}}} \times {{{\mathrm{C}}}}.$$

B is the dilution ratio of reaction system and C is the dilution ratio of the sample before measurement.

### Lipid Peroxidation (TBARS) Assay

One milliliter emulsion sample was mixed with 2.0 mL TBA reagent [17% (w/v) trichloroacetic acid and 0.8% (w/v) thiobarbituric acid dissolved in 0.1 M NaOH] and then incubated in boiling water bath for 15 min. All samples were centrifuged at 1000 g for 10 min after a rapid cooling using ice-water bath. The supernatant of samples was collected to determine the absorbance at 532 nm. The concentration of TBARS was determined by the standard curve of 1,1,3,3-tetraethoxypropane^29^.

### Determination of hydroperoxide concentration

Lipid hydroperoxides content was determined by a simple and sensitive spectrophotometric method (FOX method)^30^. Peroxides oxidize Fe^2+^ to Fe^3+^ in the acid solution., Fe^3+^-xylenol orange complex was formed in the presence of xylenol orange, which has a characteristic absorption at 560 nm. Samples were mixed with 100 μL methanol in the test tubes with a vortex mixer for 3–6 s, then 900 μL FOX reaction mixtures (25 mM H_2_SO_4_, 100 μM xylenol orange, 250 μM FeSO_4_, and 4 mM BHT in 90% (v/v) methanol) was added and incubated for 30 min at room temperature. The absorbance was determined at 560 nm.

### Statistical analysis

Data are expressed as the average of triplicate measurements or experiments. One-way ANOVA or *t*-test were used to determine significant differences. Significance was determined at *p* < 0.05 while highly significance was determined at *p* < 0.01.

## Supplementary information

Supplymentary Tables

## Data Availability

The authors declare that all data supporting the findings of this study are presented in the article. The raw data are available on request.
