# Supplementary material for: Effects of peroxidase and superoxide dismutase on physicochemical stability of fish oil-in-water emulsion
Source: NPJ Sci Food. 2022 Jun 23;6:31. doi: 10.1038/s41538-022-00146-2 (PMC9226006; doi:10.1038/s41538-022-00146-2)
Supplement: Supplementary file 1 — Supplymentary Tables [file 41538_2022_146_MOESM1_ESM.docx]

Supplementary Table 1. Granular property of FOE in the presence of HRP on Day 7

| HRP Concentration(μM) | 0 | 0.16 | 0.32 | 0.48 | 0.64 | 0.8 | 1.6 | 2.4 |
| --- | --- | --- | --- | --- | --- | --- | --- | --- |
| Size (nm) | 111.9 | 115 | 113 | 92.07 | 539.4 | 453.2 | 432.7 | 318.5 |
| PDI | 0.157 | 0.184 | 0.172 | 0.261 | 0.507 | 0.499 | 0.466 | 0.235 |
| Size Distribution Peak 1(nm) | 133.9 | 144.4 | 139.8 | 85.62 | 47.76 | 88.16 | 143.1 | 407.1 |
| Size Distribution Peak 2(nm) | 0 | 0 | 0 | 0 | 0 | 581.3 | 715 | 0 |
| Count Rate (kcps) | 105764.4 | 98558.6 | 94863 | 26168.4 | 16987.7 | 8307.5 | 10882.7 | 21357.6 |

Supplementary Table 2. Granular property of FOE in the presence of SOD on Day 7

| SOD Concentration(μM) | 0 | 0.16 | 0.32 | 0.48 | 0.64 | 0.8 | 1.6 | 2.4 |
| --- | --- | --- | --- | --- | --- | --- | --- | --- |
| Size (nm) | 120.4 | 121.7 | 121.4 | 122.3 | 121.6 | 121.3 | 121.9 | 119.3 |
| PDI | 0.198 | 0.197 | 0.206 | 0.206 | 0.207 | 0.177 | 0.202 | 0.195 |
| Size Distribution Peak 1(nm) | 151.6 | 150.6 | 156.1 | 154.3 | 156.1 | 156.9 | 156.9 | 151.3 |
| Size Distribution Peak 2(nm) | 0 | 0 | 0 | 0 | 0 | 0 | 0 | 0 |
| Count Rate (kcps) | 75918.9 | 72869.5 | 73110.3 | 74667.6 | 73954.6 | 71425.8 | 71425.8 | 71496.2 |
